# A Male Patient With Systemic Lupus Erythematosus Presenting With Fulminant Hepatitis

**DOI:** 10.4021/gr343w

**Published:** 2011-11-20

**Authors:** Sukran Erten, Aylin Demirezer Bolat, Fatma Ebru Akin, Osman Ersoy

**Affiliations:** a Departments of Rheumatology, Ataturk Education and Research Hospital, Ankara, Turkey; b Departments of Gastroenterology, Ataturk Education and Research Hospital, Ankara, Turkey

**Keywords:** Systemic lupus erythematosus, Hepatitis, Liver failure, Multiorgan failure

## Abstract

Systemic lupus erythematosus (SLE) is an autoimmune disease with unknown etiology. Although liver dysfunction is rare in SLE, 25 - 50% of patients may develop abnormal liver functions. Here, we described a case presented as fulminant hepatitis and diagnosed as SLE.

## Introduction

Systemic lupus erythematosus (SLE) is an autoimmune disease characterized by multisystem involvements, including the gastrointestinal system (GIS) [1]. Although the etiology is unknown, it is believed to develop in genetically predisposed individuals in the presence of an environmental factor [2]. GIS manifestations include gastro esophageal reflux, dysphagia, abdominal pain, constipation, diarrhea, fecal perforation, intestinal pseudo-obstruction, perforation, and hemorrhage [1]. Although clinical liver dysfunction is rare in SLE, 25-50 % of patients may develop abnormal liver function tests. After exclusion of secondary causes, hepatitis due to SLE occurs in 3 - 5% of cases [3]. We describe a case who was presented as fulminant hepatitis and diagnosed as SLE.

## Case report

A 45 year-old male patient was admitted to gastroenterology department with a three-week-history of fatigue, loss of appetite and musculoskeletal pain. His past medical and family history were not significant. Physical examination revealed minor apthous ulcer on the tongue and icteric sclera. There was pain on knees, ankles and muscles of leg during the examination. Initial laboratory evaluation yielded as follows: serum glutamate-oxalate transaminase (SGOT) level was 977 U/L (5 - 34), serum glutamate-pyruvate transaminase (SGPT) level was 786 U/L (5 - 49), lactate dehydrogenase (LDH) level was 364 U/L (0 - 190), gamma-glutamyl transferase (GGT) was 604 U/L (2 - 73), alkaline phosphatase(ALP) was 196 U/L (25 - 129) and creatine phosphokinase (CPK) was 183 U/L (32 - 294). The patient was diagnosed as acute hepatitis and hospitalized. Written informed consent was obtained from the patient.

Hepatitis serology was negative for hepatitis B surface antigen (HBs Ag) and hepatitis C virus (HCV). Anti HBs, Anti HBe and Anti HBc total were positive. Serum total bilirubin level was 1.1 mg/dL (0 - 0.2) and direct bilirubin level was 0.66 mg/dL (0 - 0.2). Hepatitis B virus deoxyribonucleic acid (DNA) was negative. On complete blood count, there were leukopenia (WBC 2300) and thrombocytopenia (platelet count 72 000). ESR was 12 mm/h, CRP was 11.4 mg/L (0 - 4.9), albumin 2.2 g and ferritin > 1650 ng/mL (22-322). ANA was 4 positive and homogenous. Anti double stranded DNA was greater than 200 IU/mL (0 - 20) and antimitochondrial antibody (AMA) was positive. C3 was 0.265 g/L (0.9 - 1.8) and C4 was 0.102 g/L (0.1-1.4). Direct coombs test was also positive. Abdominal ultrasonography demonstrated pericholecystic fibrosis around bile duct. Toracoabdominal CT (computerized tomography) showed bilateral pleural effusion and fluid collection at retroperitoneal region and obliteration of presacral region. Autoimmune hepatitis was suspected and hepatic biopsy was performed. Minimal lobuler inflammation and reactive changes on hepatocytes were detected. It was not diagnostic for autoimmune hepatitis. He was consulted to hematology department for bicytopenia and bone marrow biopsy demonstrated polyclonal plasma cell increase as 10% and myeloid/erythroid ratio was 3/1. The patient was diagnosed as SLE.

Due to inefficient oral intake, parenteral nutrition was started and ursodeoxyclolate was added to treatment. Systemic corticosteroid treatment was planned but at that time fever was added to patient’s complaints. Serum CRP level was increased to 109 mg/L. Blood culture was positive for gram negative bacillus Echerichia Coli. Ceftriaxone 2 g/day was prescribed. Since the fever was subsided, pulse steroid treatment as prednisolone 1 gm/day for 3 days and pulse cyclophoshamide 1 gm/day were administered at 5th day of antibiotic treatment. Since then, the general condition of the patient worsened and respiratory distress started. D-Dimer value was increased to 5892 ng/mL (0 - 500). Thorax CT and ventilation perfusion scintiography were not representative for pulmonary thromboembolism. Serum CPK level was elevated progressively to 12879 U/L, GGT was 1224 U/L, PT 26 second (11.2 - 13.2), aPTT 156 second (22 - 31), urea 230 mg/dL, creatinine 3 mg/dL and sodium was 119 mmol/L (136 - 145). Hemodialysis was performed twice but the general condition of the patient worsened. He was transferred to intensive care unit and died there one day later.

## Discussion

SLE is a multisystem autoimmune disease with a broad spectrum of clinical presentations [2]. The prevalence of SLE is much lower in males than females and male SLE patients may have a less favorable outcome [4].

Liver dysfunction in patients with collagen disease is generally mild and the prognosis is fine irrespective of the cause [5]. Although clinical liver dysfunction is rare in SLE, 25 - 50% of patients may develop abnormal liver function tests. The etiology is multifactorial and can be induced by disease activity [6]. Miller et al proposed that liver enzyme elevation correlates with SLE disease activity in a subset of patients [3].

Liver dysfunction in the presence of systemic lupus erythematosus is difficult to distinguish from autoimmune hepatitis. Histological examination of liver is useful to make differential diagnosis [5]. Lupoid hepatitis is differentiated from SLE on the basis of liver lesion being initial and dominant and is called as autoimmune hepatitis (AIH). New criteria for classification of AIH have been proposed and the two can be distinguished by clinical, serologic, and pathologic categories [3]. Liver biopsy of our patient was not in accordance with autoimmune hepatitis.

Toxic hepatitis is regarded as a cause of high levels of liver enzymes in lupus. Hepatotoxicity of aspirin, NSAIDs (non-steroid anti-inflammatory drugs), and minocycline have been reported in lupus [6]. Our patient had no history for drug or herbal medicine usage.

The pattern of liver enzyme elevation varies between patients. If hepatocyte injury predominates, increased aminotransferase levels may be the dominant finding. Elevations of alkaline phosphatase and bilirubin levels predominate in cholestatic syndromes [6]. Elevation of CPK as seen in our patient was probably due to SLE related myositis.

SLE patients could have variable GIS manifestations. They may be the initial or major presentations of SLE. In a study, 30.8% of the SLE patients presented with GIS symptoms. When the GIS presents as the initial affected system of SLE, there is likely a time lag before the clinician to realize the underlying disease. Some SLE related GIS complications may be life-threatening if not treated promptly with corticosteroids and immunosuppressants, as it was also seen in our patient. Therefore, early recognition of the disease may prevent delay of treatment [1].

Runyon et al described SLE patients with liver disease; 43 among 206 met the study inclusion criteria. Death of 3 patients from liver failure led the authors to conclude that liver involvement in SLE can be fatal [7].

SLE was associated with an increased risk of viral infections. An association between SLE exacerbations and viral infections has been documented [2]. Fulminant hepatic failure due to de novo hepatitis B is lethal, and the possibility of hepatitis B should be investigated in patients receiving steroid therapy. In particular, checking the hepatitis core and surface antibodies is necessary in Hbs Ag negative patients [5]. Our patient had no evidence of acute viral hepatitis and elevation of the liver function was related to disease activity of SLE.

In patients with liver enzyme elevation, besides the other etiologies like viral or drug induced hepatitis, autoimmune diseases like SLE should be kept in mind.
